# Application of Botulinum Neurotoxin Injections in TRAM Flap for Breast Reconstruction: Intramuscular Neural Arborization of the Rectus Abdominis Muscle

**DOI:** 10.3390/toxins13040269

**Published:** 2021-04-09

**Authors:** Kyu-Ho Yi, Hyung-Jin Lee, Ji-Hyun Lee, Kyle K. Seo, Hee-Jin Kim

**Affiliations:** 1 Wonju City Public Health Center, Wonju-Si, Kangwondo 26417, Korea; kyuho90@daum.net; 2 Department of Oral Biology, Division in Anatomy and Developmental Biology, Human Identification Research Institute, BK 21 FOUR Project, Yonsei University College of Dentistry, 50-1 Yonsei-ro, Seodaemun-gu, Seoul 03722, Korea; leehj221@yuhs.ac (H.-J.L.); jh_anatomy@naver.com (J.-H.L.); 3 Modelo Clinic, Seoul 06011, Korea; doctorseo@hotmail.com; 4 Department of Materials Science & Engineering, College of Engineering, Yonsei University, Seoul 03722, Korea

**Keywords:** myocutaneous flap, rectus abdominis muscle, botulinum neurotoxin, Sihler’s method

## Abstract

Breast reconstruction after mastectomy is commonly performed using transverse rectus abdominis myocutaneous (TRAM) flap. Previous studies have demonstrated that botulinum neurotoxin injections in TRAM flap surgeries lower the risk of necrosis and allow further expansion of arterial cross-sectional diameters. The study was designed to determine the ideal injection points for botulinum neurotoxin injection by exploring the arborization patterns of the intramuscular nerves of the rectus abdominis muscle. A modified Sihler’s method was performed on 16 rectus abdominis muscle specimens. Arborization of the intramuscular nerves was determined based on the most prominent point of the xyphoid process to the pubic crest. All 16 rectus abdominis muscle specimens were divided into four muscle bellies by the tendinous portion. The arborized portions of the muscles were located on the 5–15%, 25–35%, 45–55%, and 70–80% sections of the 1st, 2nd, 3rd, and 4th muscle bellies, respectively. The tendinous portion was located at the 15–20%, 35–40%, 55–60%, and 90–100% sections. These results suggest that botulinum neurotoxin injections into the rectus abdominis muscles should be performed in specific sections.

## 1. Introduction

Breast reconstruction after mastectomy is commonly performed using the transverse rectus abdominis myocutaneous (TRAM) flap. The TRAM flap uses an autologous myocutaneous flap consisting of skin, subcutaneous fat, blood vessels, and the rectus abdominis muscle [1]. The benefits of using autologous tissue, such as in TRAM, include superior cosmetic results and avoidance of implant-associated complications [2]. However, TRAM flap surgery still carries the risk of postoperative complications, such as tissue necrosis.

Previous studies have demonstrated that botulinum neurotoxin (BoNT) injections in TRAM flap surgeries lower the risk of necrosis and allow further expansion of arterial cross-sectional diameters [3]. The identified mechanism of action of BoNT is a prolonged inhibition of acetylcholine release in the neuromuscular junction of the rectus abdominis muscle; as a result, muscle contraction is blocked and the risk of tissue ischemia is decreased [4,5,6,7]. The main objective in reconstructive procedures is ensuring tissue survival after transplantation using pedicled or free flaps. Furthermore, muscle contractions after TRAM surgery can cause pain in patients, and BoNT injections are known to relieve pain by decreasing muscle tone [8].

Presently, the main treatment for reducing muscle contractions is through the injection of BoNT, which is recognized as one of the most effective and safest treatment options for this purpose [9,10,11]. When injecting BoNT, the dose administered should be sufficient to allow toxin buildup in the arborized neural network [8,12,13]. The effectiveness of injections that target neural arborized sections, which contain the most neuromuscular junctions, has been investigated in clinical studies on psoas major and biceps brachii muscles [14,15]. Furthermore, injections directed at sections of arborization have been demonstrated to result in significantly greater reductions in muscle volume than injections performed on conventional sites [14,15].

Clinicians should be aware of several factors when using BoNT, since high doses can cause undesirable paralysis as the toxin reaches adjacent muscles [16,17]. Furthermore, the use of high doses and repeated BoNT injections accelerates the production of antibodies, which leads to decreased toxin efficacy [16,17,18]. Consequently, BoNT must be injected into sections of neural arborization to maximize efficiency and reduce adverse effects. Previous studies have revealed the location of intramuscular arborization sections in other muscles; these sections have been suggested as the ideal sites for BoNT injections [19,20,21,22,23,24,25,26].

To date, no study has been able to pinpoint the locations of intramuscular neural arborizations in the rectus abdominis. In this study, we used the whole-mount staining method first suggested by Sihler to demonstrate intramuscular neural distributions without injuring the nerves [27,28]. The findings of this study allow for the identification of injection points for BoNT in TRAM flaps.

## 2. Results

### 2.1. Intramuscular Arborization Patterns of the Rectus Abdominis Muscle

**Figure 1:**
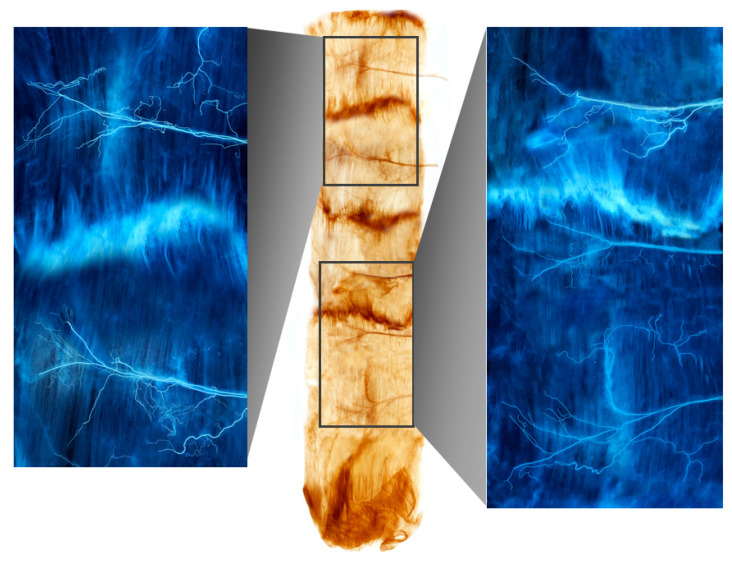
A Sihler-stained rectus abdominis muscle. Note the intramuscular arborizations shown in the enlarged panels.

Ten of the 16 rectus abdominis muscle specimens were divided into four muscle bellies. Based on the distance from the xyphoid process to the pubic crest, the arborizations of the intramuscular nerves were largest in sections 5–15%, 25–35%, 45–55%, and 70–80% from the 1st to the 4th muscle belly, respectively (Figure 1). Three specimens had the largest arborizations in the 5–15%, 30–35%, 45–55%, and 70–85% sections. Two specimens had the largest arborizations in the 10–15%, 30–35%, 45–55%, and 75–85% sections. Finally, one specimen had the largest arborizations in the 5–15%, 30–35%, 45–55%, and 75–85% sections.

### 2.2. Tendinous Portion of the Rectus Abdominis Muscle

Fourteen out of the 16 rectus abdominis muscle specimens had the tendinous portion located in the 15–20%, 35–40%, 55–60%, and 90–100% sections, relative to the distance from the xyphoid process of the sternum to the crest of the pubis. Two specimens had the tendinous portion located in the 15–20%, 35–40%, 55–65%, and 90–100% sections.

## 3. Discussion

The rectus abdominis is a flat and strap muscle that extends along the whole length of the anterior wall of the abdomen and is separated into two portions by the linea alba. Tendinous intersections subdivide each rectus abdominis muscle into smaller muscle bellies [29]. The 1st, 2nd, 3rd, and 4th muscle bellies of the rectus abdominis are innervated by the 8th, 9th, 10th, and 12th intercostal nerves, respectively, which enter the muscles from the deeper side. The 11th intercostal nerve innervates parts of the 3rd and 4th muscle bellies near the third tendon. The 8th, 9th, 10th, and 12th intercostal nerves enter the muscles at the mid parallel line of each muscle belly. The rectus abdominis has dual blood supply: the superior and inferior epigastric arteries. The TRAM flap procedure can be achieved as a free flap based on the inferior epigastric artery or pedicled flap based on the superior epigastric artery [30,31,32].

A free TRAM is wholly detached and implanted to the breast and anastomosed to internal mammary or the thoracodorsal vessels. In a pedicled TRAM, the tissues are bulkily rotated and relocated in a state of one side attached. In most of the cases, the free TRAM flaps have better results in shaping and lesser risk of insufficient blood supply than the pedicled flap.

Previous studies have suggested that TRAM flap surgery results in a severe painful symptomatology, which necessitates the use of pain medications, [33] and post-transplantation muscle contractions and spasms are considered to be the underlying causes [34,35,36]. Recently, muscle tissue changes due to contraction-induced hypoxia in the muscle have been identified, and the association of a low oxygen level with pain has been described [37]. Additionally, deposits of glycogen and resulting interstitial fibrosis in the muscles with hypoxia have been reported. Several studies revealed that BoNT infiltration of the flap muscle in breast reconstruction surgery had produced prolonged inhibition of muscle contraction and postoperative pain [38,39,40]. Schweizer et al. demonstrated that BoNT injection increased flap survival through enhanced blood flow and oxygen supply to the tissues [41,42]. Studies examining the positive effects of BoNT on tissue perfusion and flap survival from the last decade have already been published [4,42,43,44].

BoNT irreversibly blocks neurotransmitter release in the presynaptic neuron by coupling with the presynaptic cell membrane [45]. Thus, clinicians not only need to manage the toxin precisely in the target rectus abdominis muscle, but must also inject the toxin close to the site of action; specifically, in the arborized section of the muscle. Furthermore, the large amounts of BoNT into the body can lead to the creation of antibodies against the toxin, which reduces its therapeutic effect. The rectus abdominis muscle, which is a multiply-banded, superficial abdominal muscle, requires multiple injections; this increases the risk of developing complications in nearby organs. This study made several important contributions in the discovery of the ideal injection sites in the rectus abdominis to maximize the effect of BoNT while using the smallest dose possible.

Furthermore, the method of exposing the arborization patterns of intramuscular neural networks used in this study can be applied to electromyography studies of the rectus abdominis and may also find use in sports medicine, respiratory studies, and in the diagnosis of neuromuscular injuries [46,47].

**Figure 2:**
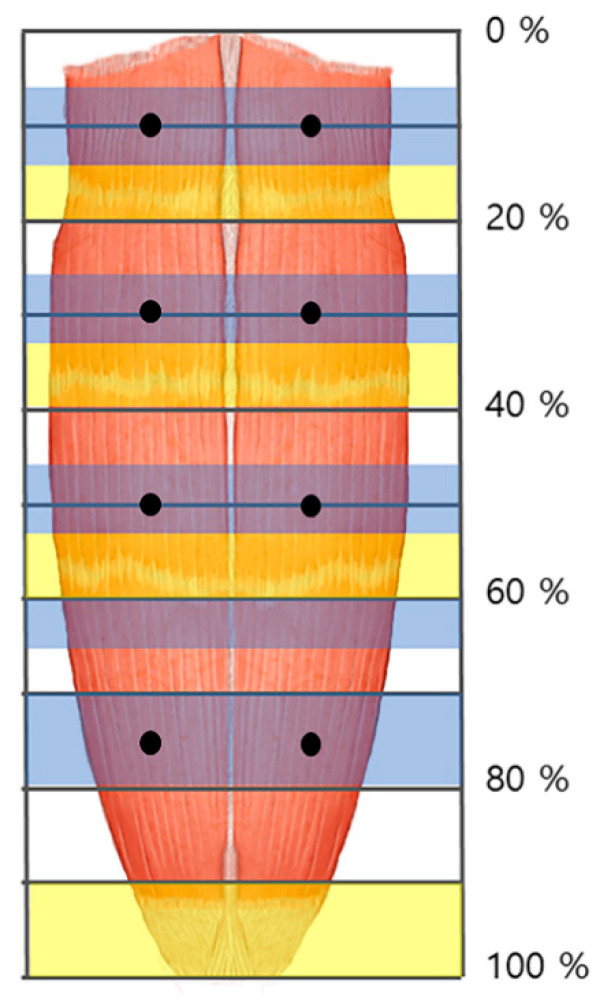
The arborized portions of the rectus abdominis muscle were located in the 5–15%, 25–35%, 45–55%, and 70–80% sections (blue shaded) from the 1st to the 4th muscle belly, respectively. The tendinous portions were located in the 15–20%, 35–40%, 55–60%, and 90–100% sections (yellow shaded). The injection points of the rectus abdominis muscle are at both sides of the midpoint at 10%, 30%, 50%, and 75% (dark dot).

We propose that practitioners perform low dose BoNT injections at several points to achieve maximum therapeutic effect while minimizing side effects (Figure 2). Furthermore, we suggest that BoNT injections and electromyography be performed in the arborized sections of the rectus abdominis muscle and to avoid the tendinous portion.

Previous studies administered amounts of BoNT injection to the rectus abdominis muscle ranging from 200–600 units with one to six injection sites [48,49,50]. However, according to the result, we recommend an injection of BoNT in pedicled flap at eight points, and free flap at the lower four points (Figure 2). Additionally, we suggest each injection dosed 25 units, since the average thickness of the rectus abdominis muscle is 10 mm [29], and BoNT usually spreads up to 2–4 cm from the injection site [13].

## 4. Materials and Methods

This study was conducted in compliance with the principles set forth in the Declaration of Helsinki. Permission and approval were obtained from the families of the cadavers before beginning the dissections. A total of 16 rectus abdominis muscles from Korean cadavers (five men and five women with a mean age of 74.6 years; range, 63–83 years) were dissected in Yonsei University medical center from May 2020 to October 2020. Modified Sihler staining was performed on all specimens to highlight the sections of arborization of the intramuscular nerves.

**Figure 3:**
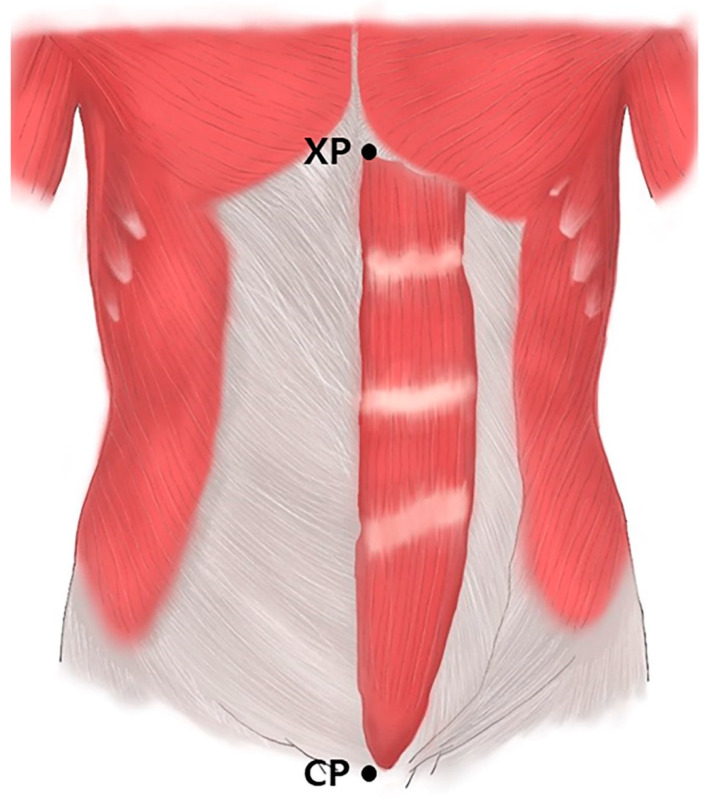
Rectus abdominis muscles. Specimens were harvested from the xyphoid process of the sternum (XP) to the crest of pubis (CP).

Prior to the dissection, the rectus abdominis muscles were aligned within their anatomical positions. The arborization patterns were tracked based on two landmarks: the xyphoid process of the sternum (0%) and the crest of the pubis (100%) (Figure 3). The muscles were divided transversely into 20 sections with each section representing a value of 5%, and the distribution of the intramuscular nerves was tracked with microscopic dissection prior to the staining procedure.

The muscle specimens underwent Sihler staining as modified by Liem and Douwe van Willigen [51]. This method includes several phases to visualize the arborization pattern of the intramuscular nerves. After staining, the muscles were measured from the xyphoid process of the sternum (0%) to the crest of the pubis (100%).

### Modified Sihler Staining

**Figure 4:**
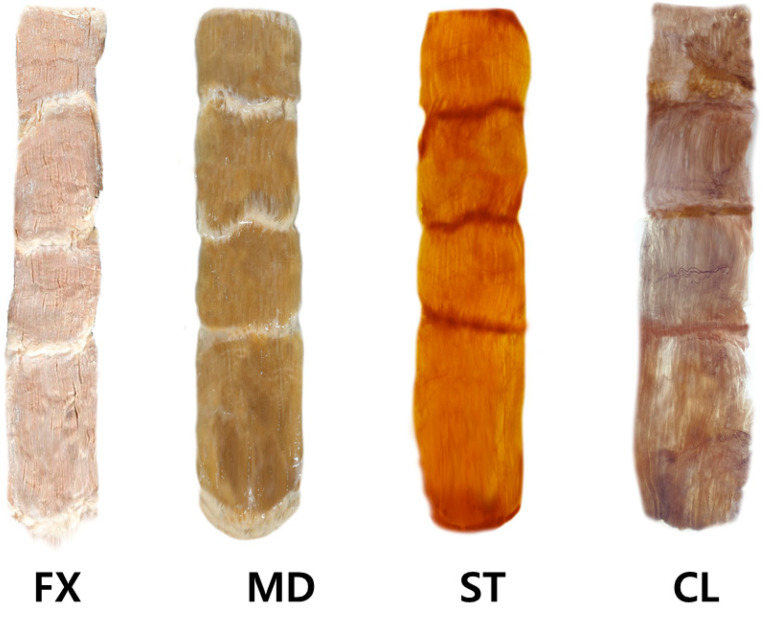
The rectus abdominis muscle underwent the modified Sihler’s method. The method consists of stages of fixation (FX), maceration and depigmentation (MD), decalcification, staining (ST), and clearing (CL).

The steps involved in performing the modified Sihler staining on the rectus abdominis muscles are presented in Figure 4, described as follows:

Fixation phase: The harvested rectus abdominis muscles were fixated for 30 days in 10% un-neutralized formalin solution. This solution was changed every time it became opaque.

Maceration and depigmentation phase: The fixed rectus abdominis muscle specimens were cleaned in pure water for an hour. Afterward, the samples were depigmented for four weeks in a solution containing hydrogen peroxide and 3% aqueous potassium hydroxide.

Decalcification phase: The macerated specimens were placed in Sihler I solution (glycerin, aqueous chloral hydrate, and glacial acetic acid) for one day.

Staining phase: After decalcification, the specimens were immediately placed in Sihler II solution (glycerin, aqueous chloral hydrate, and acetic acid) for four weeks.

Destaining phase: The stained muscle specimens were then destained in Sihler I solution for an hour.

Neutralization phase: The destained specimens were then neutralized in flowing water for half an hour and subsequently placed in 0.05% lithium carbonate for an hour.

Clearing phase: The neutralized rectus abdominis muscle specimens were finally washed with increasing concentrations (20% to 100%) of glycerin for four days.

## Data Availability

Not applicable.
